# Supplementary material for: Dual-view oblique plane microscopy (dOPM)
Source: Biomed Opt Express. 2020 Nov 18;11(12):7204–20. doi: 10.1364/BOE.409781 (PMC7747899; doi:10.1364/BOE.409781)
Supplement: Supplementary file 1 [file boe-11-12-7204-s001.pdf]

Supplemental Document

Biomedical Optics EXPRESS

Dual-view oblique plane microscopy (dOPM): supplement

HUGH SPARKS,[1] LUCAS DENT,[2] CHRIS BAKAL,[2] AXEL BEHRENS,[3] GUILLAUME SALBREUX,[4] AND CHRIS DUNSBY[1,5,*]

[1] *Photonics Group, Department of Physics, Imperial College London, London, SW7 2AZ, UK*
[2] *Dynamical Cell Systems Team, The Institute of Cancer Research, London, SW3 6JB, UK*
[3] *Cancer Stem Cell Team, The Institute of Cancer Research, London, SW3 6JB, UK*
[4] *The Theoretical Physics of Biology Laboratory, The Francis Crick Institute, London, NW1 1AT, UK*
[5] *Centre for Pathology, Imperial College London, London, SW7 2AZ, UK*
[*] *christopher.dunsby@imperial.ac.uk*

This supplement published with The Optical Society on 18 November 2020 by The Authors under the terms of the Creative Commons Attribution 4.0 License in the format provided by the authors and unedited. Further distribution of this work must maintain attribution to the author(s) and the published article's title, journal citation, and DOI.

Supplement DOI: https://doi.org/10.6084/m9.figshare.13154051

Parent Article DOI: https://doi.org/10.1364/BOE.409781

Dual-view oblique plane microscopy (dOPM): supplemental document

Hugh Sparks[1], Lucas Dent[2], Chris Bakal[2], Axel Behrens[3], Guillaume Salbreux[4], Chris Dunsby[1, 5 *]

[1]*Photonics Group, Department of Physics, Imperial College London, London, SW7 2AZ, UK*
[2]*Dynamical Cell Systems Team, The Institute of Cancer Research, London, SW3 6JB, UK*
[3]*Cancer Stem Cell Team, The Institute of Cancer Research, London, SW3 6JB, UK*
[4]*The Theoretical Physics of Biology Laboratory, The Francis Crick Institute, London, NW1 1AT, UK*
[5]*Centre for Pathology, Imperial College London, London, SW7 2AZ, UK*

** christopher.dunsby@imperial.ac.uk*

Figure S1. Mechanical design for the mount used to hold mirrors M2 and M5.

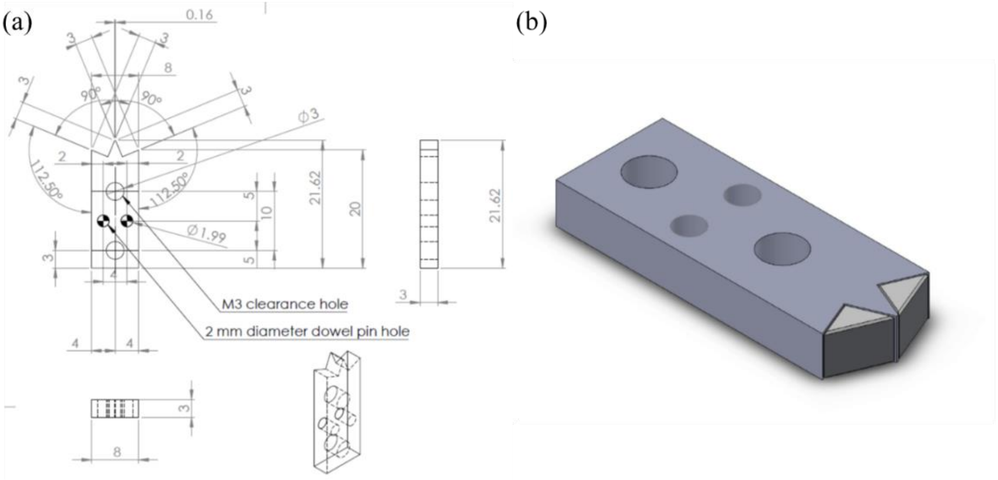

Figure S1. (a) CAD drawing prism mount made from aluminum. (b) 3D rendering of prism mount with a pair of Thorlabs dielectric coated mirrored prisms (MRA03-E02, Thorlabs).

Figure S2 Further explanation of operation of fold mirror M6 in Fig. 2.

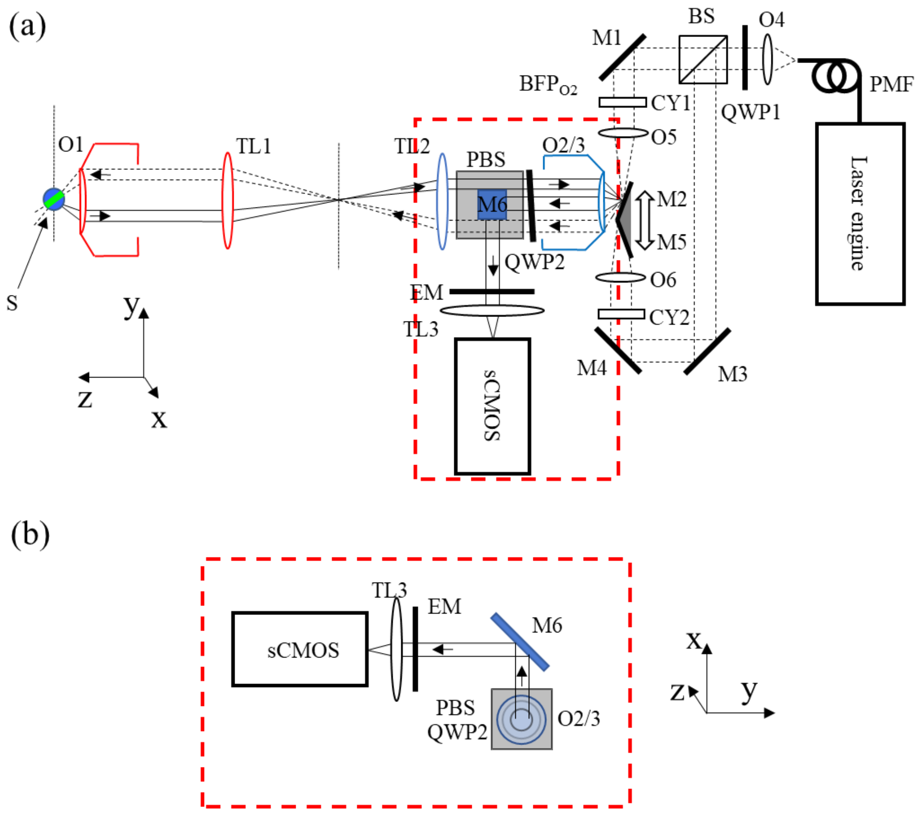

Fig. S2. Dual-view OPM (dOPM) optical configuration, which is based around a remote-refocussing setup folded by mirrors M2 or M5 about the remote microscope objective O2/3. (a) schematic of the optical setup for acquiring View 1 (See Fig.1 and Section 2.1 for more details in the main text). In (a) O, microscope objective; BFP, back focal plane; TL, tube lens; M, mirror; CY, cylindrical lens; QWP, quarter-wave plate; BS, non-polarising beam splitter; PBS, polarising beam splitter; and EM, emission filter. M2 and M5 are held on a common mount and translated together in the direction shown by the white double-ended arrow. The PBS is oriented so that the reflected fluorescence emission comes vertically up out of the plane of the page and is then reflected into the horizontal plane by M6. (b) shows how the emission is directed out of the plane of the schematic shown in (a) and towards the camera (sCMOS) using fold mirror M6.

Figure S3. Detail on how the motion of M2 causes illumination and detection to be scanned in the sample.

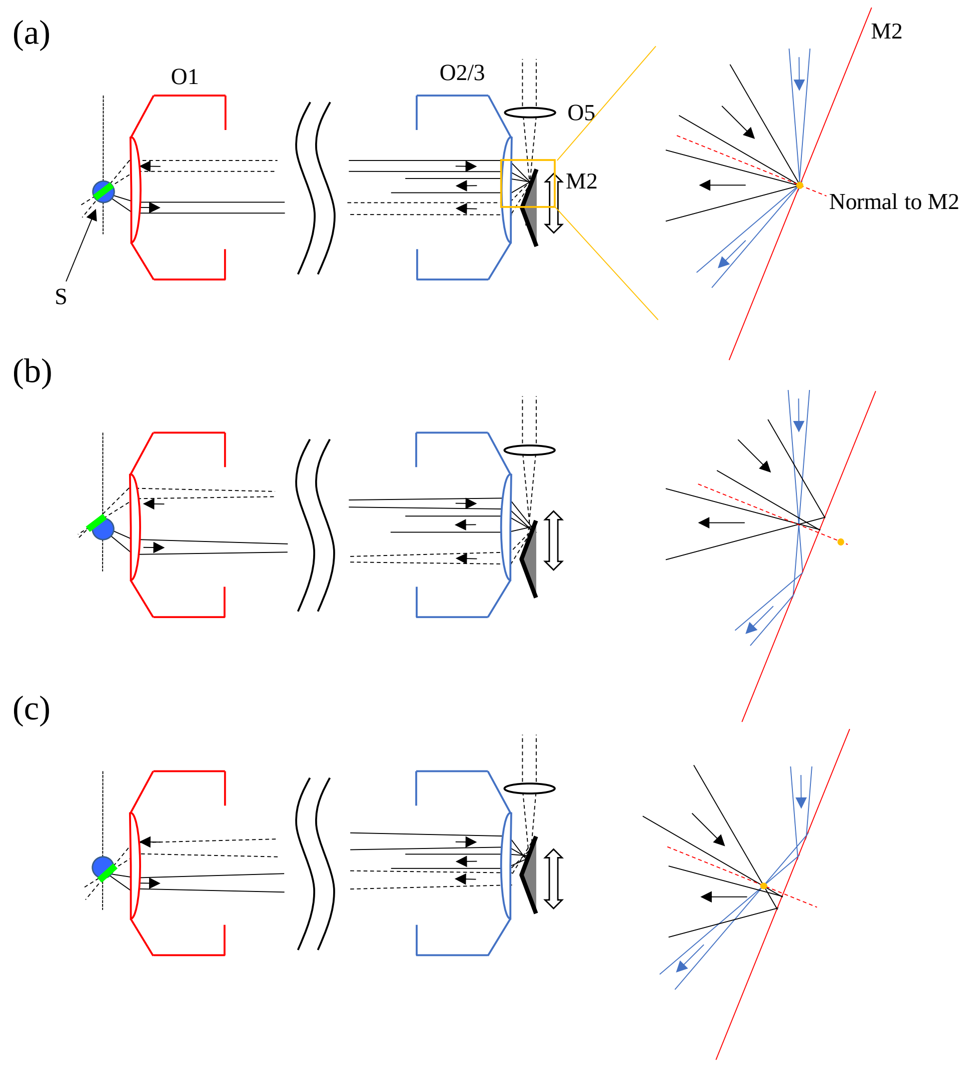

Figure S3. (a) ray diagram when illumination light-sheet waist is aligned to surface of mirror M2. (b) diagram for when M2 is translated below the position shown in (a) causing the illumination beam waist to appear to come from a point to the right of M2 (orange dot). (c) diagram for when M2 is translated above the position shown in (a) causing the illumination beam waist to appear to come from a point to the left of M2 (orange dot). The motion of M2 causes the image of the illumination beam waist in the sample to be translated along the direction normal to the surface of M2.

Figure S4 Uniformity of fluorescence excitation/detection across the swept volume for View 1 and View 2.

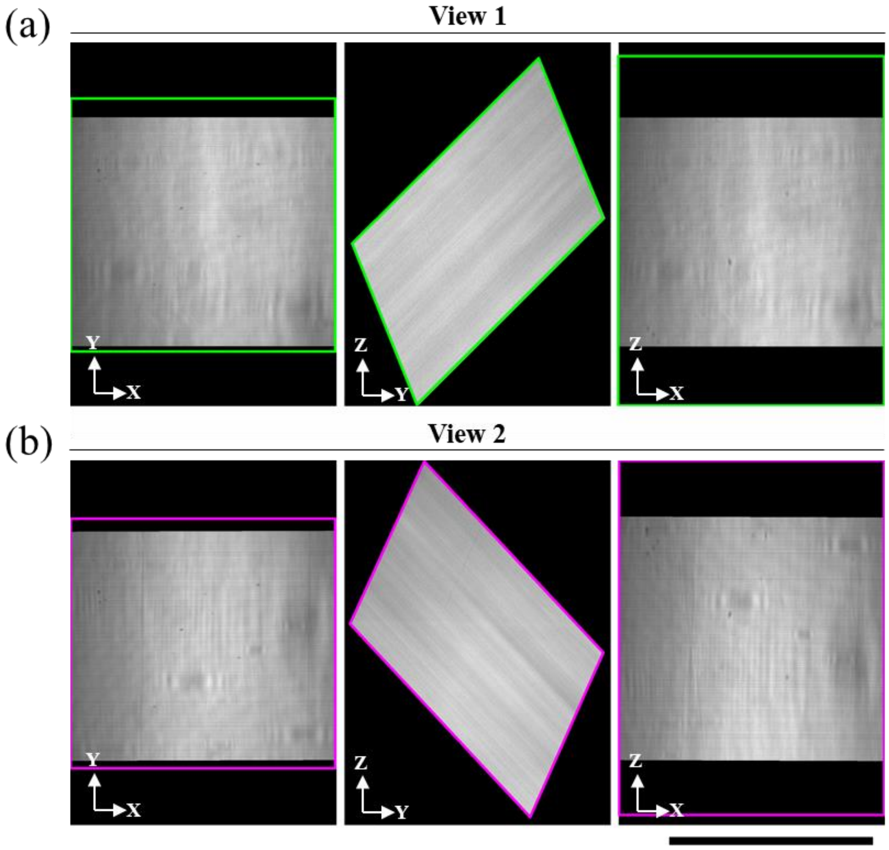

Fig. S4. Central orthogonal planes through volume of fluorescein dye 30 µM in aqueous solution. Montage (a) shows values for View 1 from X-Y, Y-Z, and X-Z perspectives. The green lines highlight the edge of the scanned volume from each perspective. Montage (b) shows equivalent values for View 2 and the magenta lines highlight the edge of the scanned volume from each perspective. The scale bar is 200 µm and applies to all images.

Figure S5. Spatial variation in point spread function along the x,y,z axes for bead volume shown in Fig. 3(b)-(d).

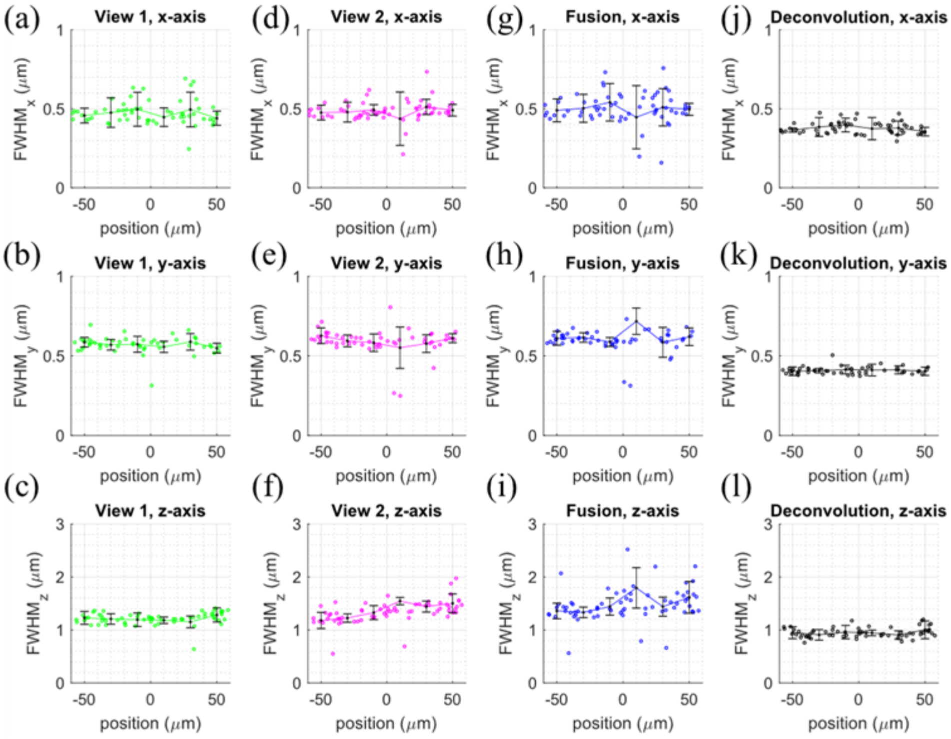

Fig. S5. Fluorescent bead full-width half maximum (FWHM) values plotted as a function of bead spatial position within the central 120(x)×120(y) ×120(z) μm^3 volume indicated by the yellow square of Fig. 3(a). The volume contained 200 nm beads embedded in agarose (See Section 2.3 in main text for methods and Fig.3 in Section 3.2 for further details). Each plot includes the data points along with a median and interquartile range values for 20 µm wide bins across each axis. Plots (a)-(c) show values for View 1, (d)-(f) show values for View 2, (g)-(i) show values for fusion of the two views and (j)-(l) show values for deconvolution of the two views.

Figure S6. 3D Fourier transform of fluorescent bead volume shown in Fig. 3(b)-(d).

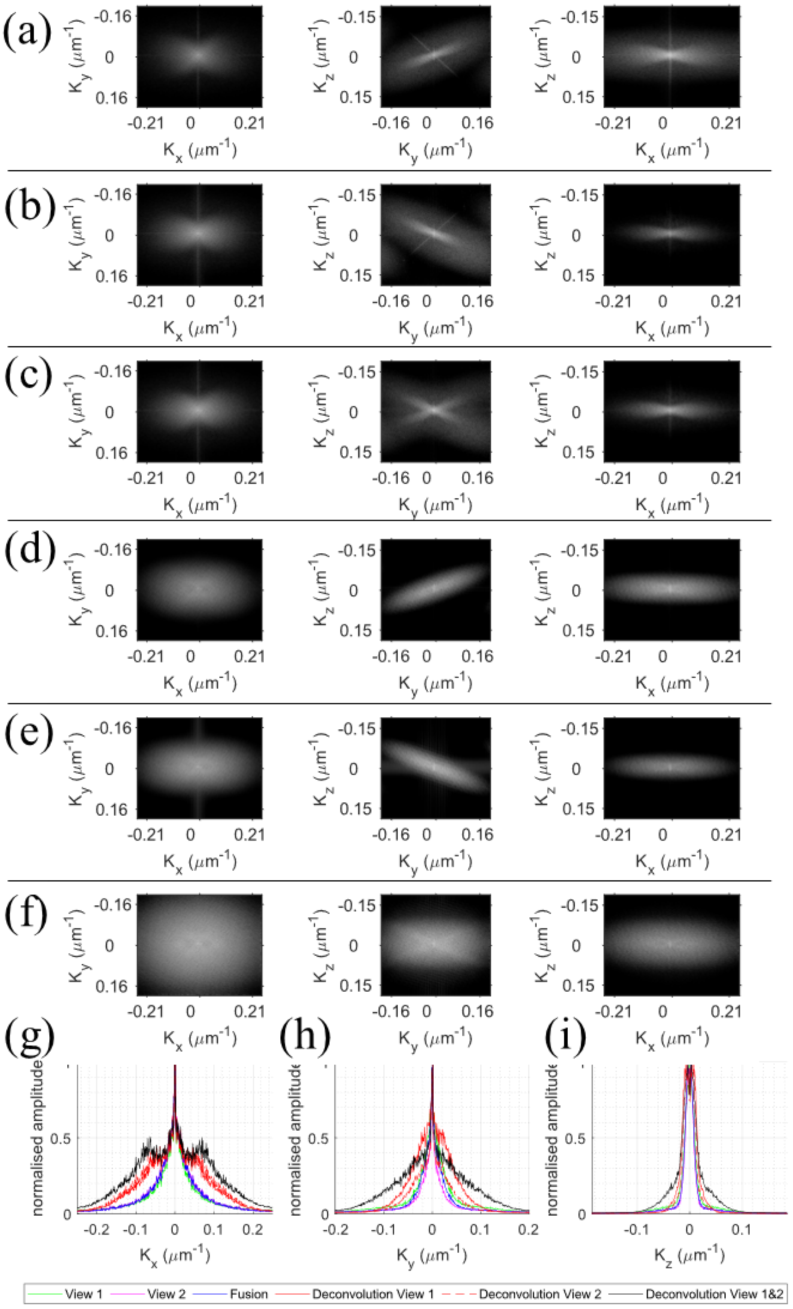

Fig. S6. Central orthogonal planes from the 3D Fourier transform of the central 120(x)×120(y)×120(z) um^3 volume of beads indicated by the yellow square in Fig. 3(a). The volume contained 200 nm fluorescent beads embedded in agarose (See Section 2.3 in main text for methods and Fig. 3 in Section 3.2 for further details). Montage (a) shows from left to right, K_x-K_y, K_y-K_z, K_x-K_z values for View 1 on a log scale and (b) shows equivalent values for View 2. Montage (c) shows values for fusion of the two views, (d) shows values for deconvolution of View 1, (e) show values for deconvolution of View 2 and (f) shows values for two-view deconvolution of View 1 & 2. Plots (g) to (i) show central orthogonal line profiles through for each OTF. For all data the zero-frequency component was removed to remove the effect of uniform background fluorescence from the bead volume. The line profiles consisted of binning a rectangular volume 11×11 pixels (0.0083×0.0083 μm^{-2}) wide to reduce noise.

Figure S7. 3D Fourier transform of spheroid volume shown in Fig. 5.

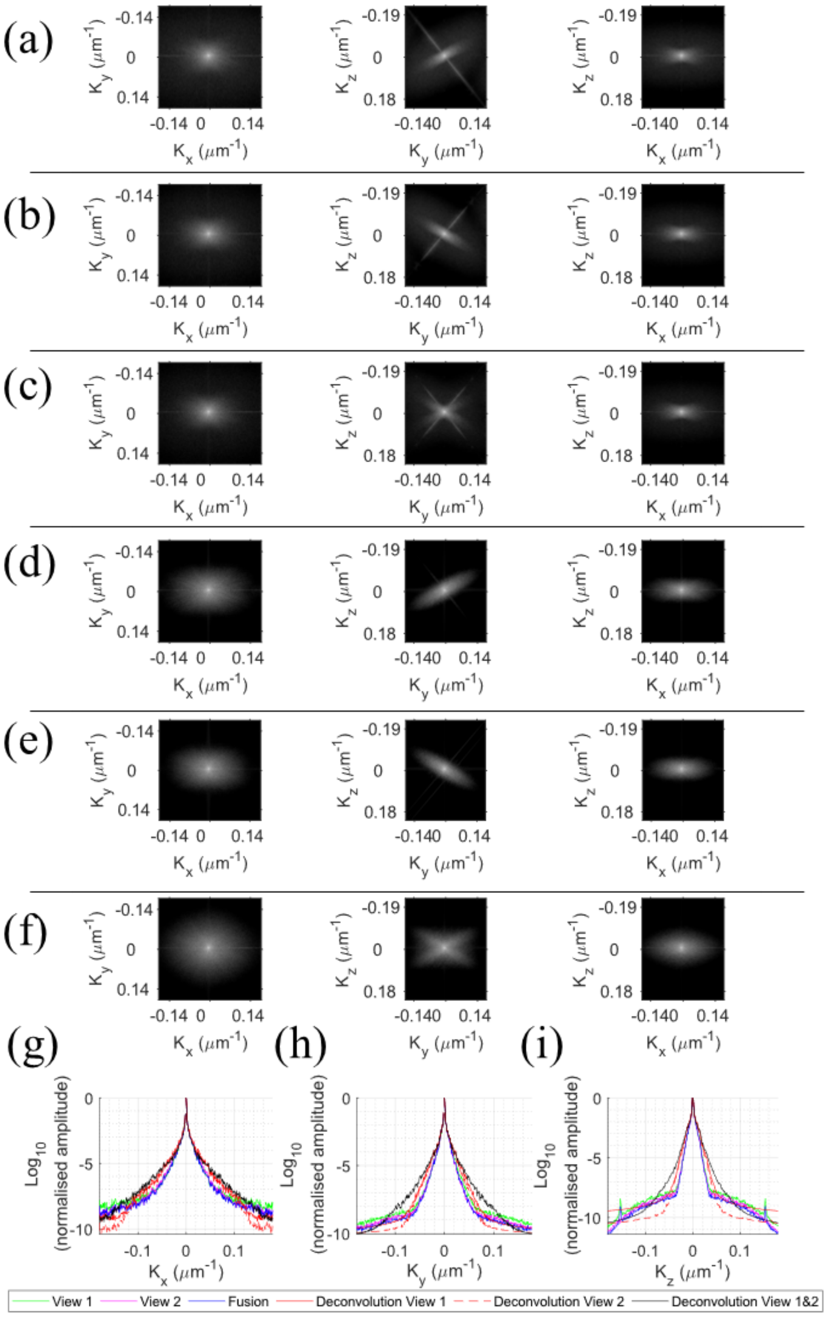

Fig. S7. Central orthogonal planes from the 3D Fourier transform of the central 120(x)×120(y)×120(z) μm^3 volume containing a fixed spheroid of WMs cells embedded in Matrigel and where Alexa Fluor™ 488 Phalloidin fluorescently labels actin (See Section 2.5 in main text for methods and Fig.5 in Section 3.3 for further details). Montage (a) shows from left to right, K_x-K_y, K_y-K_z, K_x-K_z values for View 1 on a log scale and (b) shows equivalent values for View 2. Montage (c) shows values for fusion of the two views, (d) shows values for deconvolution of View 1, (e) show values for deconvolution of View 2 and (f) shows values for two- view deconvolution of View 1 & 2. Plots (g) to (i) show central orthogonal line profiles through for each OTF. For all data shown here the zero-frequency component was removed. The line profiles consisted of binning a rectangular volume 11×11 pixels (0.0083×0.0083 μm^{-2}) wide to reduce noise.
