# Supplementary material for: Dual-view oblique plane microscopy (dOPM)
Source: Biomed Opt Express. 2020 Nov 18;11(12):7204–20. doi: 10.1364/BOE.409781 (PMC7747899; doi:10.1364/BOE.409781)
Supplement: Related texts

# Spotlight

**Published:** 2020-12-01

**Authors:** Shakil Rehman

Optical microscopy by illuminating a specimen with a thin sheet of light offers advantages like no other microscopic technique. Oblique plane microscopy (OPM) is an innovative form of light sheet microscopy utilizing a high numerical aperture microscope objective for both fluorescence excitation and collection providing video-rate 3D visualization of live biological specimens without photo bleaching and phototoxicity. The authors of this article published in <em>Biomedical Optics Express</em>, present a further development of the OPM technique as a folded dual-view oblique plane microscopy (dOPM) that enables them to obtain two orthogonal views of a sample by translating a pair of tilted mirrors in what they call a refocusing space. The mirrors are moved using a single actuator for scanning the light sheet and detection plane through the sample as well as selecting between the two orthogonal views. This leads to image processing of the two views with reduced sample induced artifacts, for example, and to the use of deconvolution for improved resolution as compared to a single view OPM.<br/><br/>
